# Multi-target pharmacological mechanisms of *Iris tectorum* Maxim. (CSG) against tonsillitis and dyspepsia: A network pharmacology approach

**DOI:** 10.1097/MD.0000000000044516

**Published:** 2025-09-26

**Authors:** Shujie Hao, Yamei Yuan, Xiangming Fang, Weidong Ye

**Affiliations:** a College of Nursing, Anhui University of Chinese Medicine, Hefei, Anhui Province, P.R. China; b Key Laboratory of Geriatric Nursing and Health, Anhui University of Chinese Medicine, Hefei, Anhui Province, P.R. China; c College of Traditional Chinese Medicine, Anhui University of Chinese Medicine, Hefei, Anhui Province, P.R. China.

**Keywords:** Chinese herbal, drugs, dyspepsia, ethnopharmacology, tonsillitis

## Abstract

*Iris tectorum* Maxim. (CSG) is a notable traditional Chinese medicinal herb recognized for its broad pharmacological activities, indicating potential therapeutic effects in treating tonsillitis and dyspepsia. This study employed a network pharmacology approach to elucidate the shared mechanisms of CSG in the treatment of tonsillitis and dyspepsia. First, potential targets of CSG were predicted. Second, disease-related genes for tonsillitis and dyspepsia were retrieved from public databases. Third, a protein–protein interaction network was constructed and analyzed using gene ontology and Kyoto encyclopedia of genes and genomes. Finally, molecular docking was performed to validate key targets. The main active components of CSG included tectoridin, tectorigenin, rhamnocitrin, and rhamnazin, with 1148 key targets identified. We found 14 shared target genes between CSG and the 2 diseases. Using Cytoscape for analysis, we created a network diagram with 66 nodes. Molecular docking revealed that serine/threonine-protein kinase 1, mitogen-activated protein kinase 1, epidermal growth factor receptor, vascular endothelial growth factor A, and phosphoinositide-3-kinase regulatory subunit 1 are likely involved in CSG’s multi-target effects. The potential pathways of CSG in treating tonsillitis and dyspepsia involve the regulation of endogenous deoxyribonuclease activity, nucleotide excision repair, and DNA damage recognition, among other biological processes. These findings suggest that CSG’s potential effects on tonsillitis and dyspepsia may involve multi-compound, multi-target, and multi-pathway mechanisms, providing preliminary mechanistic insights for future research.

## 1. Introduction

Tonsillitis, which belongs to the category of “arthralgia of throat” in Traditional Chinese Medicine (TCM),^[1]^ is a common disease of upper respiratory tract infection. As key immune organs, the tonsils are constantly exposed to airborne and ingested pathogens, making them particularly vulnerable to infection.^[2]^ The global prevalence of chronic tonsillitis ranges from 5% to 12%, with notably higher rates in children and adolescents.^[3]^ Modern studies have shown that its etiology is multifactorial, involving bacterial or viral infections, immune dysfunction, and environmental influences.^[4]^ External factors such as poor dietary habits, irregular lifestyle, and poor general health status can compromise the immune defense of the throat, increasing both the incidence and severity of tonsillitis.^[5,6]^ Its clinical treatment primarily involves antibacterial and anti-inflammatory medications. While effective for acute symptoms, prolonged use can result in to adverse effects, including gastrointestinal disturbances, allergic responses, and the development of antibiotic resistance. Moreover, in recurrent cases, these treatments often fail to prevent relapse, and some patients may eventually require surgery. These limitations highlight the need for alternative or complementary approaches.^[7–9]^ Dyspepsia is induced by gastric dynamic disorder, which belongs to the category of “wan pi,” “wei tong,” and “cao za” of TCM. With an incidence of about 20% to 30% in China and 8% to 23% in Asian countries,^[10,11]^ dyspepsia is associated with various factors, including *Helicobacter pylori* infection, structural abnormalities of the upper gastrointestinal tract, impaired gastrointestinal motility, mucosal inflammation, psychological conditions such as anxiety and depression, and lifestyle-related factors such as diet, medication use, and stress.^[12–14]^ Recent studies have highlighted that both inflammatory cell infiltration and heightened inflammatory cytokine expression are potentially pivotal in the development of dyspepsia.^[15,16]^ In summary, dyspepsia is a complex condition with a multifactorial pathogenesis involving various biological and psychosocial factors, requiring a comprehensive management approach. TCM offers diverse therapeutic approaches for dyspepsia, including complex herbal formulations, external therapies, and individualized syndrome differentiation.^[17,18]^ Further high-quality evidence is essential to elucidate the underlying mechanisms and validate its clinical efficacy.

CSG, the dried root of *Iris tectorum* Maxim., is commonly used as a genuine regional drug in Sichuan province.^[19]^ As a newly added medicinal material in the 2005 edition of *Pharmacopoeia of the People’s Republic of China*, with the effect of detoxification and pharynx, CSG can clear phlegm and eliminate hematoma and has a wide range of clinical applications.^[20]^ *Iris tectorum* Maxim. was first recorded in *Sheng Nong’s Herbal Classic*, known as “Important medicine for throat pain.” The quality standard of the Chinese Pharmacopoeia in 2020 was to determine the content of the rhizome by high performance liquid chromatography.^[21]^ Modern research shows that the chemical components of CSG are mainly isoflavones and their glycosides, phenylquinones, and triterpenoids.^[22]^ Pharmacological studies have demonstrated that the total flavonoids, the major bioactive constituents of CSG, exert various therapeutic effects, including antitussive, expectorant, antipyretic, analgesic, anti-inflammatory, antibacterial, and antiviral activities.^[23]^ The ethanol extract of CSG also shows significant anti-inflammatory, antitumor, antioxidant, lipid-lowering, and free radical scavenging properties.^[24]^ Among its active components, tectoridin is not only the primary bioactive compound but also a key quality control marker of CSG, exhibiting notable anti-inflammatory, estrogen-like, hepatoprotective, and antioxidant effects.^[25]^ CSG flavonoid capsules developed by the team of Professor Xu Xuemin and the Xiaoji Tongbian Capsules for gastrointestinal heat constipation in Guizhou Magic Pharmaceutical all take total flavonoids as the main component.^[26]^ Despite empirical evidence supporting the therapeutic efficacy of CSG’s active components in managing both tonsillitis and dyspepsia, the underlying molecular mechanisms remain unclear, particularly in relation to the TCM principle of “homotherapy for heteropathy.” Previous studies have generally focused on a single disease and lacked integration with TCM theory. In this study, we employed a unique methodological approach that integrates modern pharmacology with TCM theory, using network analysis to bridge theoretical concepts and experimental validation.

“Homotherapy for heteropathy” is interpreted by the TCM theory as “For different diseases, the same treatment method can be adopted if the pathogenesis is similar.”^[27]^ Although the diseased regions of tonsillitis and indigestion are different from clinical symptoms, a certain correlation exists between the 2 pathogenic factors. Therefore, the same pathogenic mechanism may accord with the theory of “different diseases” in “homotherapy for heteropathy.” The network pharmacology is based on the “disease-gene-target-drug” advantage to establish an association network between drug composition, target point and disease, which indicates the intervention and influence of drugs on the disease network. Thus, it is suitable for the study of the relationship between multiple compositions, pathways and targets of TCM, which helps clarify the complex mechanism action of TCM.^[28]^ Hence, from the perspective of TCM theory regarding “homotherapy for heteropathy,” this study investigated the mechanisms by which CSG addresses tonsillitis and dyspepsia through network pharmacological analysis. We hypothesize that *Iris tectorum* Maxim. (CSG) exerts therapeutic effects on both diseases by modulating common molecular targets and biological pathways, thereby providing a scientific basis for the TCM concept of treating distinct disorders with a unified method.

## 2. Materials and methods

**Figure 1.**
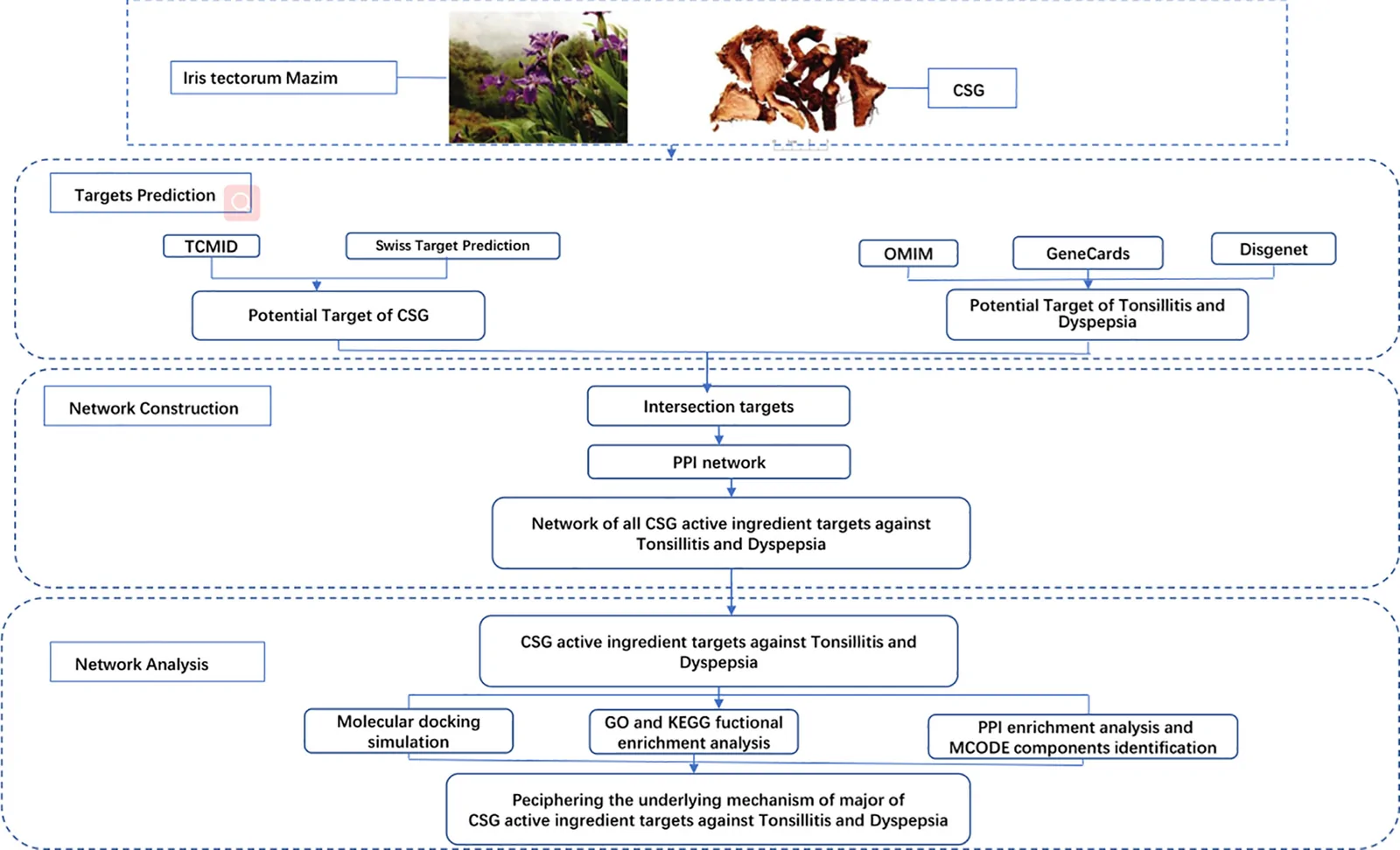
The workflow of this study.

Computational approaches were employed to identify the bioactive compounds of CSG and their corresponding pharmacological targets. Candidate genes implicated in tonsillitis and dyspepsia were systematically screened. To investigate potential interactions, a comprehensive target network was constructed. Subsequent functional analyses, including pathway enrichment and protein–protein interaction (PPI) mapping, were conducted to delineate the molecular mechanisms underlying CSG’s effects. Network pharmacology further facilitated the identification of key molecules and signaling cascades potentially mediating therapeutic outcomes. A schematic overview of the experimental design is presented in Figure 1.

### 2.1. Collection of total flavonoids composition and target points of CSG

Relevant literature was consulted and the main chemical components of CSG were collected using the Traditional Chinese Medicine Integrated Database (https://ngdc.cncb.ac.cn/databasecommons/database/id/437, Center for Bioinformatics, Peking University, China). Potential pharmacological targets of the identified compounds were predicted using Swiss Target Prediction (http://www.swisstargetprediction.ch/, Swiss Institute of Bioinformatics, Switzerland), with a probability threshold > 0. Compounds without predicted targets were excluded, and the names of all remaining targets were standardized using the UniProt database (https://www.uniprot.org/, UniProt Consortium, Europe).

### 2.2. Collection of disease genes

“Tonsillitis” and “dyspepsia” were taken as keywords in the Online Mendelian Inheritance in Man (https://omim.org/, Johns Hopkins University, Baltimore), GeneCards (https://www.genecards.org/, Weizmann Institute of Science, Israel), and DisGeNET (https://www.disgenet.org/, IMIM and UPF, Spain) databases to retrieve disease-associated targets. The common targets between the total flavonoids of CSG and the 2 diseases were summarized.

### 2.3. Analysis of the “active component-target-disease” network

The PPI network of CSG total flavonoids treating tonsillitis and dyspepsia was constructed using BisoGenet plug-in (Institute of Biomedical Engineering, Chinese Academy of Medical Sciences, China) in the software Cytoscape 3.7.0 (Cytoscape Consortium, San Diego). The purpose was to obtain a target regulation network diagram of the total flavonoids of CSG in treating the 2 diseases directly and indirectly. The CytoNCA plug-in (The University of Tokyo, Japan) was used to screen the target regulation network. The screening condition “Degree ≥ 2 * median” was applied in both rounds of analysis. For module analysis, the molecular complex detection clustering algorithm was applied using the ClusterViz plug-in (Institute of Systems Biology, Seattle),^[29]^ with the parameter K-Core set to 4. All other parameters not specifically mentioned were maintained at their default settings.

### 2.4. Gene ontology (GO) and Kyoto encyclopedia of genes and genomes (KEGG) pathway enrichment analysis

GO and KEGG pathway enrichment analyses were conducted for targets after molecular complex detection module analysis by the ClueGO plug-in (version 2.5.5; Institute of Genetics, University of Cologne, Germany) the software Cytoscape 3.7.0 (Cytoscape Consortium, San Diego).^[30]^ The threshold of *P* ≤ .05 was set, and all other parameters were maintained at their default settings.

### 2.5. Molecular docking

Download compounds SDF structure from the Pubchem database (https://pubchem.ncbi.nlm.nih.gov/, National Center for Biotechnology Information, Bethesda), use Chem3D (version 20.0; PerkinElmer, Springfield) energy minimization is saved as a mol2 format, and use Autodock Tools (version 1.5.6; The Scripps Research Institute, La Jolla) opened the ligand molecules, hydrogenation, charge, root detection of ligand, rotatable bond search and definition, and saved as a PDBQT file. Relevant targets were input on the Uniprot website (https://www.uniprot.org/, UniProt Consortium, Europe), and human-derived, high-resolution eutectic proteins with drug molecules were selected as the proteins for docking. The structure of the protein was downloaded from the RCSB Protein Data Bank (https://www.rcsb.org, Research Collaboratory for Structural Bioinformatics, Piscataway), the water molecules and ligand were deleted with PyMOL (version 2.5; Schrödinger, New York), opened in Autodock tools 1.5.6 (The Scripps Research Institute, La Jolla) by adding all the hydrogen atoms, calculating the Gasteiger charge, define it as a receptor and save it as a PDBQT file. The coordinates of the original ligand were used as the molecular docking coordinates. For proteins without ligands, a Gridbox was set to cover the entire protein. In order to improve docking accuracy, Exhaidency was set to 24. Default values are used for all parameters unless otherwise stated. In this study, AutoDock Vina (version 1.1.2; The Scripps Research Institute, La Jolla) was used for semiflexible docking, and the conformation with the best affinity was selected as the final docking conformation. The conformation with the lowest docking binding energy was selected for docking binding mode analysis. Discovery Studio (version 2020; Dassault Systèmes BIOVIA, San Diego) was used for force analysis, and PyMOL (version 2.5; Schrödinger, New York) was used for plotting, so as to visualize the results.

To assess the strength of interaction between small molecules and proteins, the binding energy was calculated. A binding energy <0 kcal·mol^−1^ indicates spontaneous binding, and a lower binding energy corresponds to stronger binding affinity.

## 3. Results

### 3.1. Collection of total flavonoids and their targets in CSG

By Traditional Chinese Medicine Integrated Database and other databases combined with literature mining, candidate target criteria screened were high-credibility proteins, and components without corresponding targets were removed. The screening results were as follows: The total flavonoids of CSG have 18 chemical compositions and 1148 targets (Table 1). These chemical compositions include tectoridin, tectorigenin, dichotomitin, iridin, irigenin, irisflorentin, irilone, genistein, iristectorin A, iristectorigenin A, iristectorin B, dihydrokaempferide, rhamnocitrin, isorhamnetin, rhamnazin, dinatin, luteolin, and isovitexin. Among them, tectoridin, tectorigenin, rhamnocitrin, rhamnazin, dinatin, luteolin, and isorhamnetin can interact with more than 100 target proteins, which may be the material basis for the treatment of tonsillitis and dyspepsia.

**Table 1:**
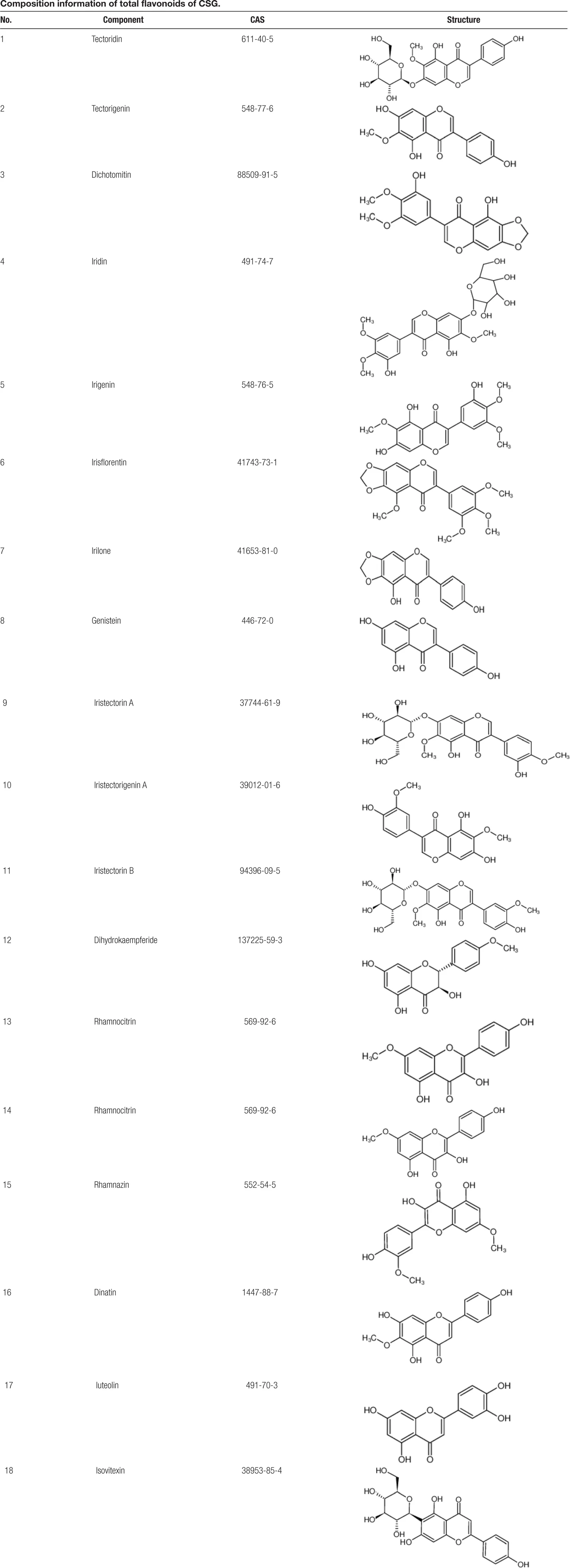
Composition information of total flavonoids of CSG.

### 3.2. Collection of targets for tonsillitis and dyspepsia

**Figure 2.**
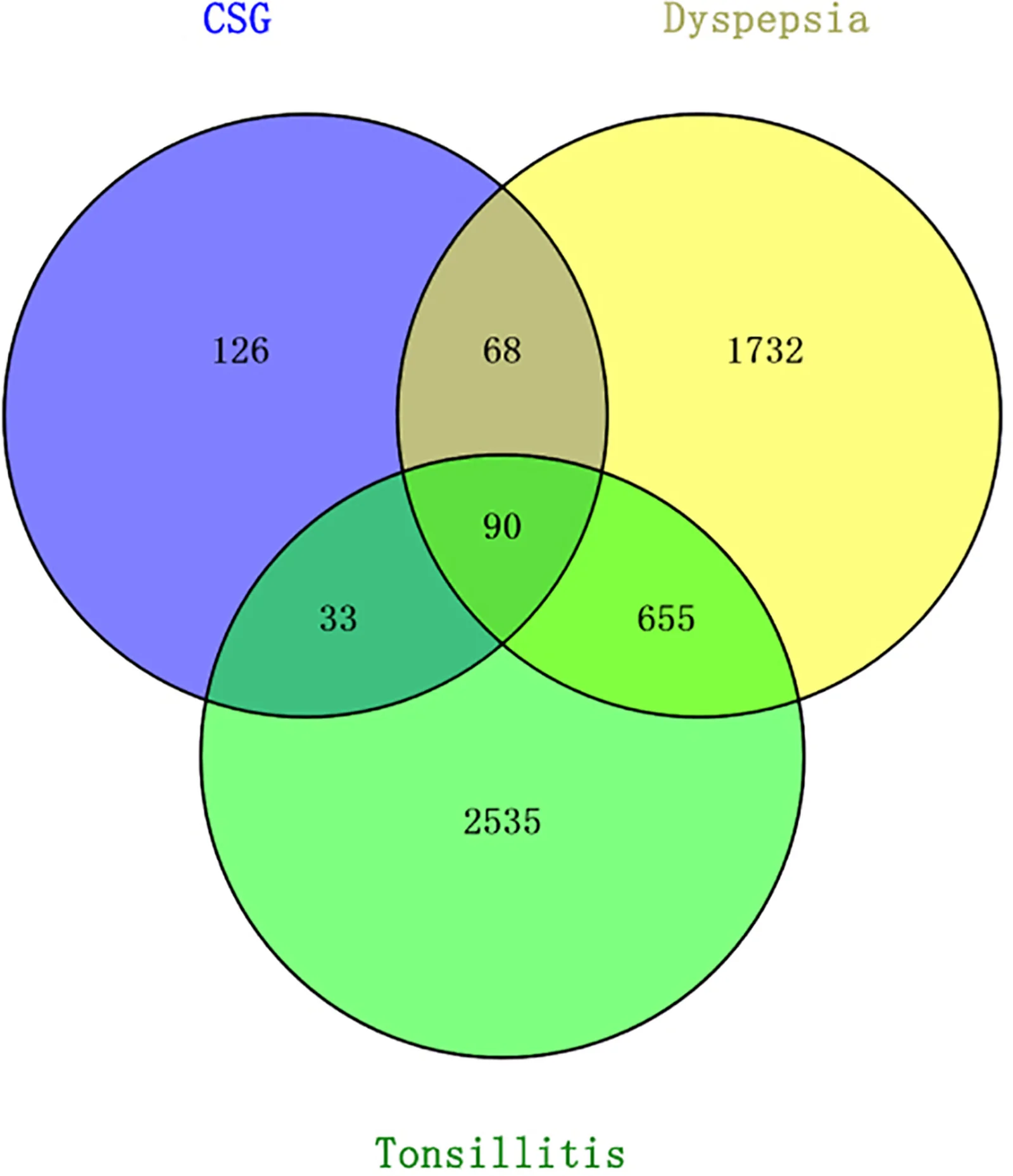
The Venn diagram shows that CSG shared 90 potential targets with known components of the pathological course related to tonsillitis and dyspepsia.

According to Online Mendelian Inheritance in Man, GeneCards, and DisGeNET databases, 3313 and 2545 disease targets have an association with tonsillitis and dyspepsia, respectively. The targets of the 2 diseases are matched with the target of total flavonoids and the main active component of CSG, respectively. A total of 123 and 158 targets of total flavonoids exist in the treatment of tonsillitis and dyspepsia. The total flavonoids of CSG have 90 common targets in the treatment of tonsillitis and dyspepsia (Fig. 2).

### 3.3. Analysis of the “active component-target-disease” network

**Figure 3.**
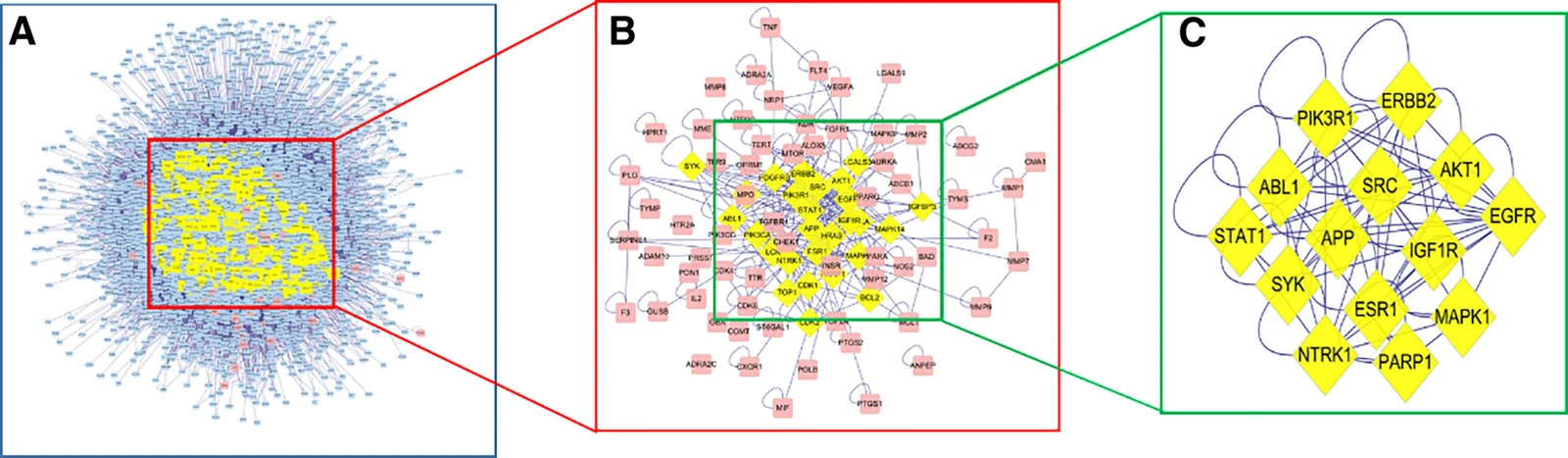
(A) The protein–protein interaction (PPI) network of targets of CSG in treating tonsillitis and dyspepsia. (B) The Cytonca plug-in was used to filter the network for the first time. (C) The PPI network of the core targets of CSG in treating tonsillitis and dyspepsia.

The PPI network of total flavones of CSG for treating tonsillitis and dyspepsia was constructed to get 3661 direct or indirect targets. A total of 119,492 relationships were observed between these targets. After filtering, 90 targets and 292 interaction relationships were obtained in the first round of analysis with a screening degree threshold of ≥68. The second round of screening yielded 14 core targets and 66 interaction relationships (Fig. 3).

**Figure 4.**
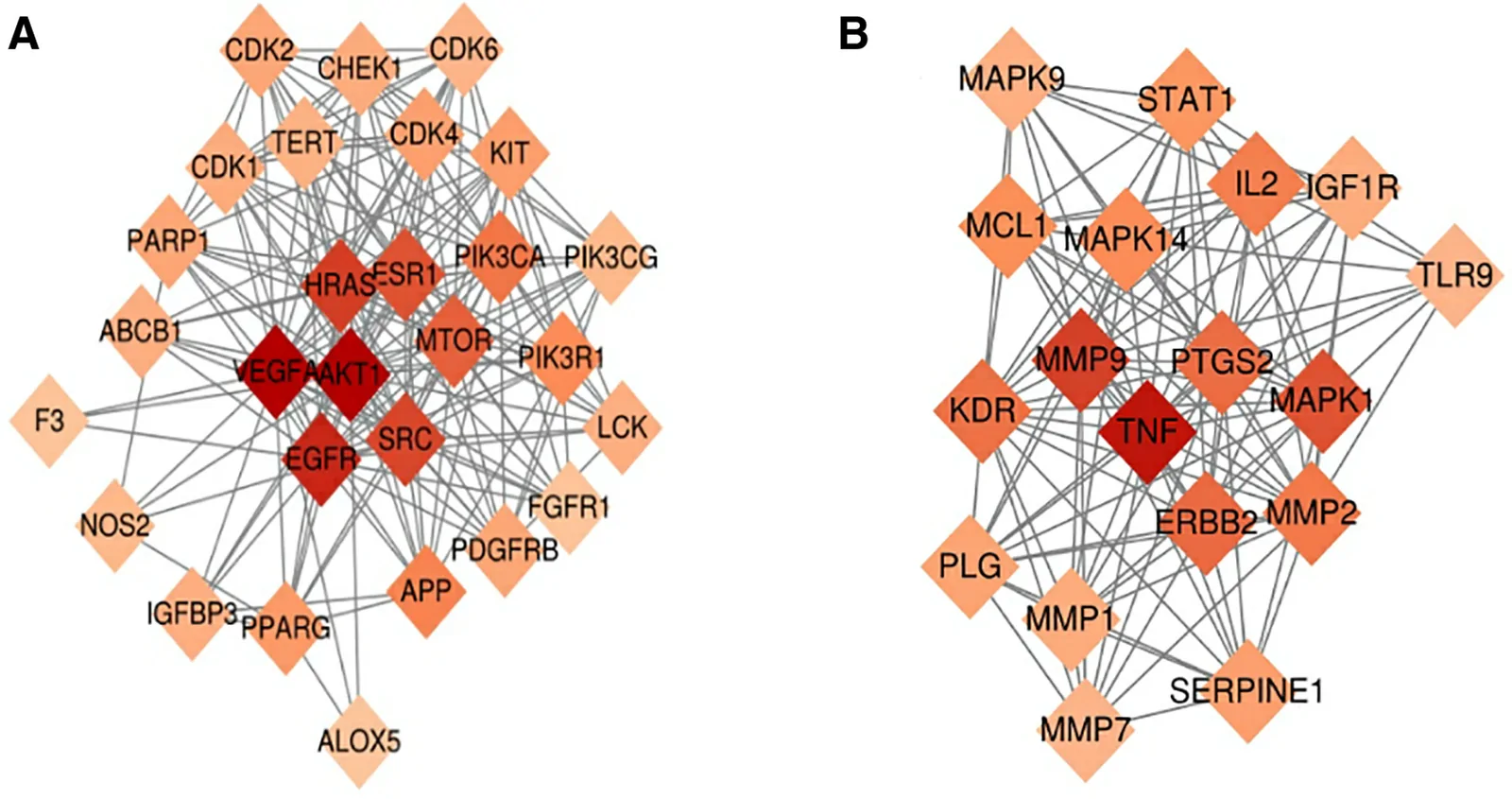
(A) and (B) Modular analysis was carried out by the MCODE algorithm in Clusterviz, and the results showed that the modules of score > 4 were sorted into 2 categories according to the score. MCODE = molecular complex detection.

Information on the 14 key targets of CSG in the treatment of tonsillitis and dyspepsia is shown in Table 2. It can be seen that these targets are primarily associated with processes such as cell proliferation and differentiation, gene transcriptional regulation, and immune responses. Among these 14 key targets, serine/threonine-protein kinase 1 (AKT1), phosphoinositide-3-kinase regulatory subunit 1 (PIK3R1), and epidermal growth factor receptor (EGFR) exhibited the highest values in both betweenness centrality and degree, highlighting their pivotal roles in the network. Furthermore, modular analysis revealed that 2 modules scored >4 and could be categorized into 2 clusters according to their topological features. The results of different modules are shown in Figure 4.

**Table 2:** Fourteen key targets information table of CSG in the treatment of tonsillitis and dyspepsia.

Number	Gene	Protein	Biological activity of targeted proteins
1	PIK3R1	Phosphoinositide-3-kinase regulatory subunit 1	Regulating the insulin metabolism
2	ERBB2	ErbB-2	Mediating cell signal transduction processes
3	ABL1	ABL proto-oncogene 1	Tyrosine kinase, mainly involved in cell differentiation, division and stress response, etc
4	SRC	Kinase Src	Encoding tyrosine protein kinase
5	AKT1	Kinase	Regulating the glucose metabolism
6	STAT1	Signal transducer and activator of transcription 1	Transcriptional Activator
7	APP	Amyloid precursor protein	Amyloid precursor protein
8	IGF1R	Insulin-like growth factor receptor	Mediating insulin-like growth factor 1
9	EGFR	Epidermal growth factor receptor	Regulating cell growth and differentiation
10	SYK	Spleen tyrosine kinase	Regulating the immune and inflammatory responses
11	ESR1	Estrogen receptor	Regulating cell proliferation and differentiation
12	MAPK1	Mitogen-activated protein kinase 1	Regulating cell growth, survival and differentiation
13	NTRK1	Neurotrophic tyrosine kinase 1	Encoding the prothrombin receptor kinase
14	PARP1	Poly [ADP-ribose] polymerase 1RA-C-alpha serine/threonine-protein	Regulating cell proliferation, differentiation and apoptosis

EGFR = epidermal growth factor receptor, MAPK1 = mitogen-activated protein kinase 1, PIK3R1 = phosphoinositide-3-kinase regulatory subunit 1.

### 3.4. GO and KEGG enrichment analyses

**Figure 5.**
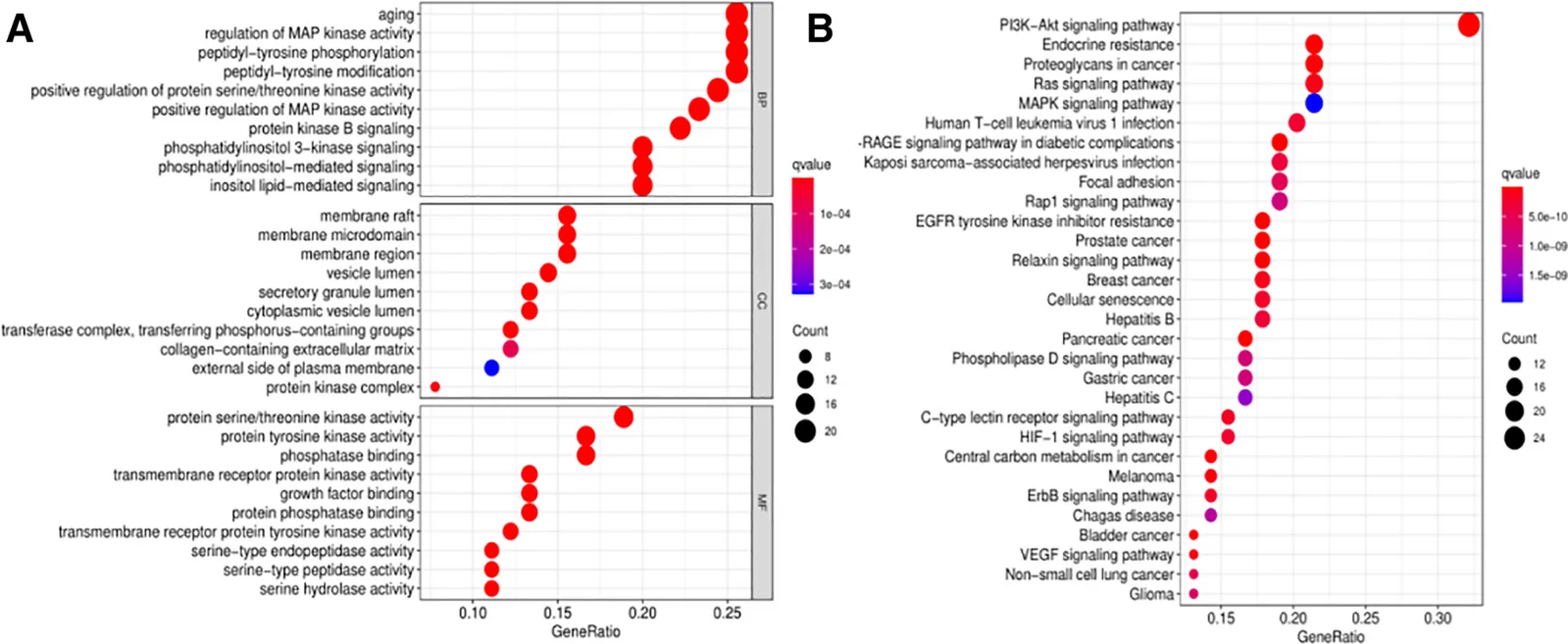
(A) The GO functional classificationof the core targets. The *x*-axis shows the enrichment scores of these terms, and the *y*-axis shows significantly enriched GO categories of the target genes. (B) The KEGG functional classification of the core targets. The *y*-axis shows the significantly enriched KEGG pathways of the target genes, and the *x*-axis shows the level of enrichment. The size of the dot indicates the number of target genes in the pathway, and the color of the dot reflects the *q* value. GO = gene ontology, KEGG = Kyoto Encyclopedia of Genes and Genomes.

**Figure 6.**
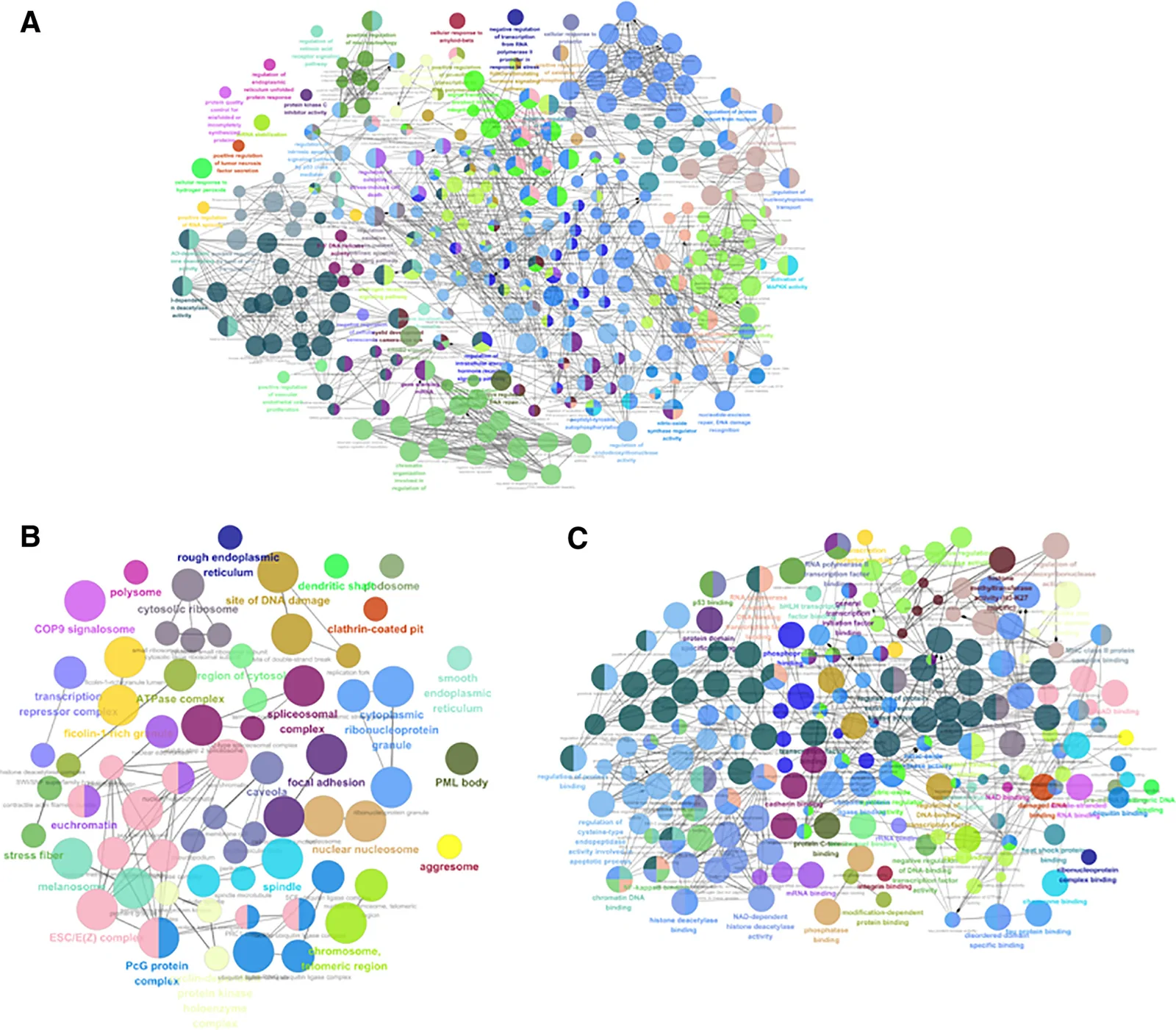
(A) Enrichment analysis of core targets of CSG in treating tonsillitis and dyspepsia (biological process). The total flavone of CSG involves 1902 biological process in treating tonsillitis and dyspepsia. (B) Enrichment analysis of core targets of CSG in treating tonsillitis and dyspepsia (cellular component). (C) Enrichment analysis of core targets of CSG in treating tonsillitis and dyspepsia (molecular function).

**Figure 7.**
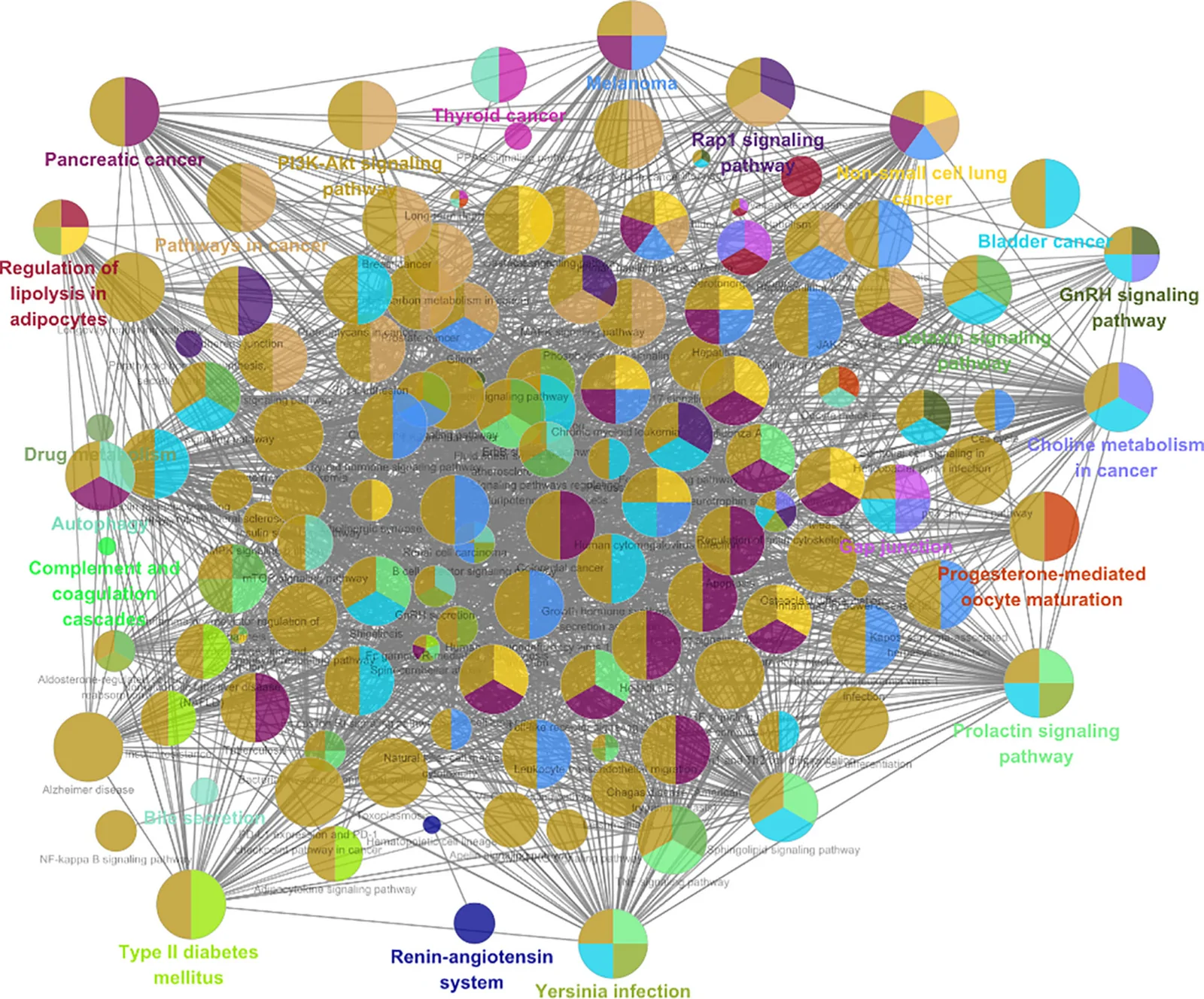
Kyoto encyclopedia of genes and genomes (KEGG) pathways. The chart shows an overview of the analysis with enriched processes and pathways.

A total of 14 targets were obtained after the target regulatory network diagram of CSG total flavonoids for the direct and indirect treatment of 2 diseases was screened twice. GO and KEGG enrichment analyses of the potential targets for CSG’s bioactive constituents in tonsillitis and dyspepsia were carried out through Metascape. The top 30 enriched terms from both analyses were classified into 2 functional clusters, as illustrated in Figure 5. GO analysis function shows that the total flavones of CSG involve 1902 biological process in the treatment of tonsillitis and dyspepsia. They are possibly related to the regulation of endogenous deoxyribonuclease activity, the repair of nucleotide excision, the identification of DNA damage and other biological process (Fig. 6A). In addition, they involve 166 cell components and are associated with cellular components such as adhesion, cell-basal junction and transcriptional regulatory complexes (Fig. 6B). They involve 183 molecular functions and are related to molecular functions such as ubiquitin protein ligase binding, ubiquitin-like protein ligase binding and DNA binding transcription factor binding (Fig. 6C). Pathway enrichment analysis identified 186 signaling pathways, with the main pathways being phosphoinositide 3-kinase/protein kinase B signaling pathway (PI3K/Akt), Rap1, and tumor necrosis factor (TNF) signaling pathways (Fig. 7).

### 3.5. Molecular docking of the active ingredient to the core target

**Figure 8.**
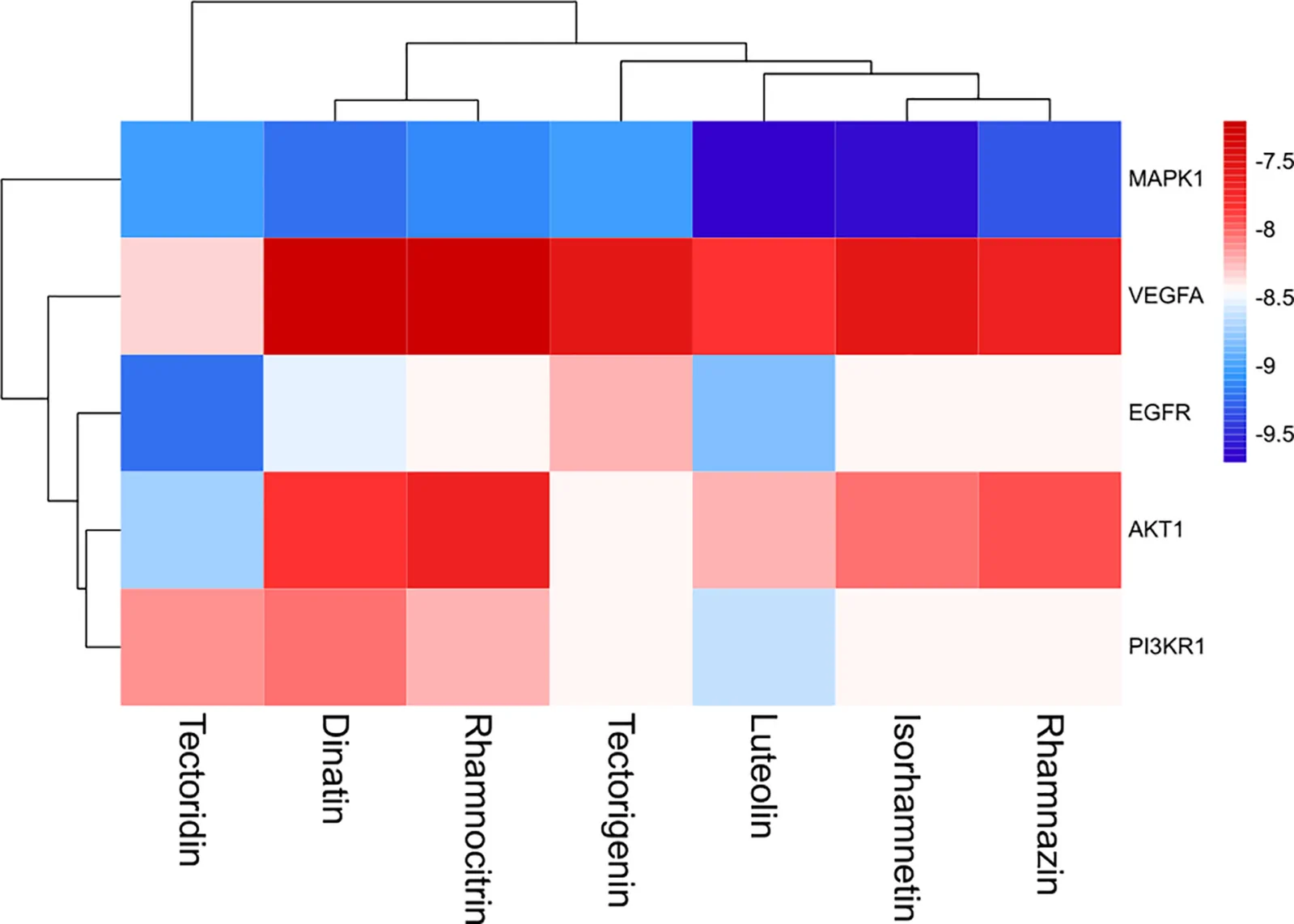
Heat map of docking results.

**Figure 9.**
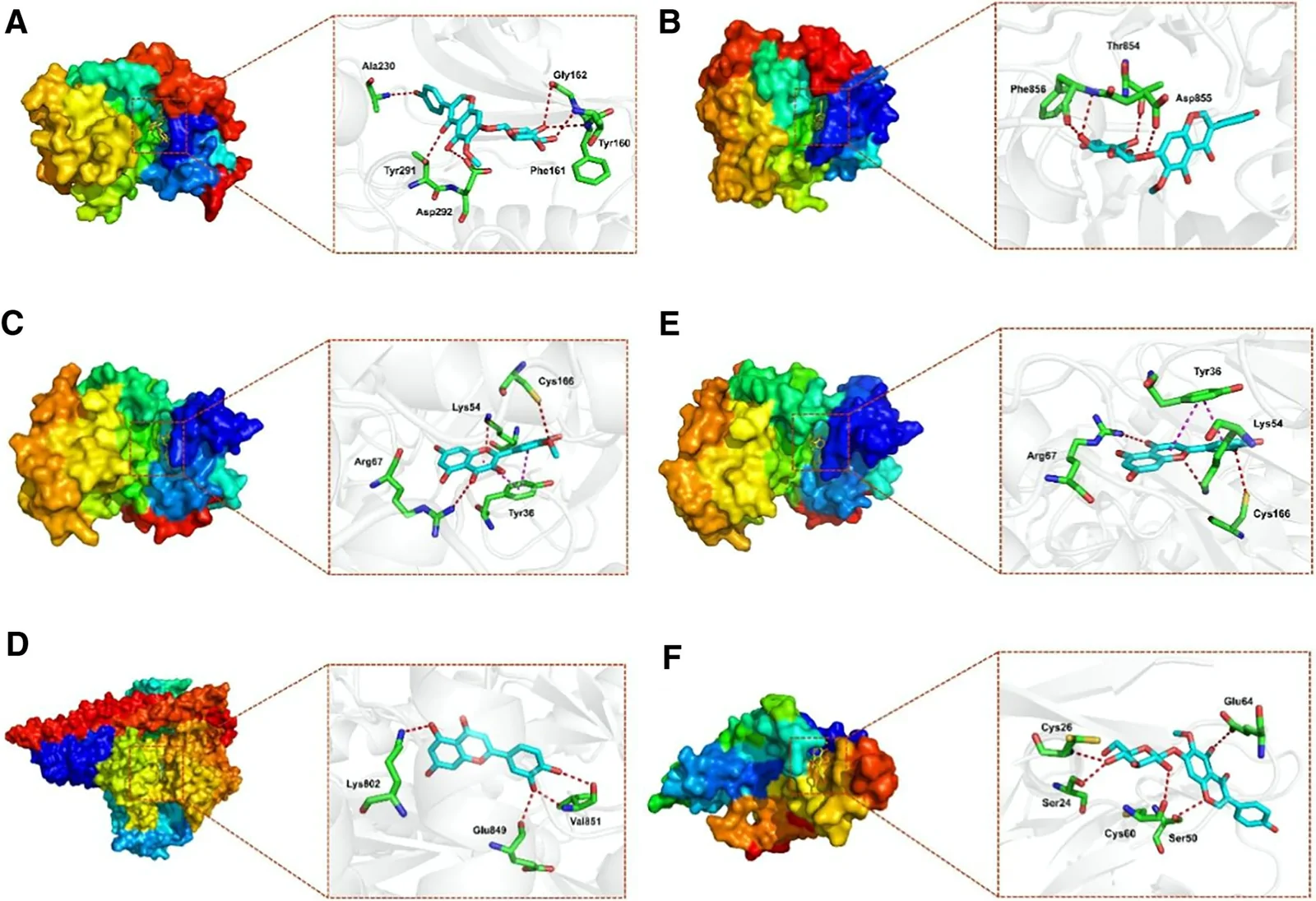
(A) Tectoridin-AKT1 interaction pattern: The binding energy of Tectoridin to AKT1 protein is −8.7 kcal/mol, forming 7 hydrogen bonds with amino acid residues Tyr160, Phe161, Gly162, Ala230, Tyr291, and AsP292. Leu156 tectoridin molecules formed hydrophobic interaction with amino acid residues Leu156, Val164, Ala177, Glu234, and Met281. (B) Tectoridin EGFR interaction pattern: the binding energy of tectoridin molecule to EGFR protein was −9.2 kcal/mol, forming 4 hydrogen bonds with amino acid residues Thr854, Asp855, and Phe856. Tectoridin molecules form hydrophobic interaction with amino acid residues Leu718, Val726, Ala743, Lys745, and Leu844. (C) Isorhamnetin–MAPK1 interaction pattern: The binding energy of Isorhamnetin to MAPK1 protein is −9.6 kcal/mol, forming 3 hydrogen bonds with amino acid residues Lys54, Arg67, and Cys166, and 2 π–π interactions with Tyr36. Isorhamnetin molecules formed hydrophobic interaction with amino acid residues Tyr36, Val39, Ile56, Glu71, and Asp167. (D) Luteolin–MAPK1 interaction pattern: the binding energy of Luteolin to MAPK1 protein is −9.7 kcal/mol, forming 3 hydrogen bonds with Lys54, Arg67, and Cys166, and 2 π–π interactions with Tyr36. Luteolin molecules formed hydrophobic interaction with amino acid residues Tyr36, Ile56, Glu71, and Asp16. (E) Luteolin–PIK3R1 interaction pattern: the binding energy of luteolin to PIK3R1 protein is −8.6 kcal/mol, forming 4 hydrogen bonds with Lys802, Glu849, and Val851. Luteolin molecules formed hydrophobic interaction with amino acid residues Ile848, Met922, and Ile932. (F) Tectoridin–VEGFa interaction pattern: the binding energy of tectoridin to VEGFa protein was −8.3 kcal/mol, forming 5 hydrogen bonds with amino acid residues Ser24, Cys26, Ser50, Cys60, and Glu64. Tectoridin molecules formed hydrophobic interaction with amino acid residues Ile46 and Glu64. AKT1 = serine/threonine-protein kinase 1, EGFR = epidermal growth factor receptor, MAPK1 = mitogen-activated protein kinase 1, PIK3R1 = phosphoinositide-3-kinase regulatory subunit 1.

The binding energies of major compounds (tectoridin, tectorigenin, rhamnocitrin, rhamnazin, dinatin, luteolin, and isorhamnetin) with potential targets of total flavonoids of CSG were as low as −7.5 kcal·mol^−1^ (1 cal ≈ 4.186 J), indicating favorable binding affinity. The Iridaceae components of CSG showed good binding activity with potential targets, as detailed in Table 3. According to docking results (Fig. 8), isorhamnetin exhibited strong affinity for mitogen-activated protein kinase 1 (MAPK1); luteolin showed strong affinity for MAPK1 and PIK3R1; and tectoridin demonstrated strong binding with AKT1, EGFR, and vascular endothelial growth factor A (Fig. 9).

**Table 3:** Docking score.

Gene	Dinatin	Isorhamnetin	Luteolin	Rhamnazin	Rhamnocitrin	Tectoridin	Tectorigenin
AKT1	−7.8	−8	−8.2	−7.9	−7.6	−8.7	−8.4
EGFR	−8.5	−8.4	−8.8	−8.4	−8.4	−9.2	−8.2
MAPK1	−9.2	−9.6	−9.7	−9.3	−9.1	−9	−9
PI3KR1	−8	−8.4	−8.6	−8.4	−8.2	−8.1	−8.4
VEGFA	−7.2	−7.5	−7.8	−7.6	−7.2	−8.3	−7.5

EGFR = epidermal growth factor receptor, MAPK1 = mitogen-activated protein kinase 1, VEGFA = vascular endothelial growth factor A.

## 4. Discussion

The primary aim of this study was to systematically explore the molecular targets and mechanisms underlying the therapeutic effects of *Iris tectorum* Maxim. (CSG) on tonsillitis and dyspepsia through network pharmacology and molecular docking analyses. Our findings identified flavonoids as key active compounds and highlighted core targets including AKT1, PIK3R1, and EGFR, which may mediate the effects of CSG via multiple signaling pathways such as PI3K/Akt, Ras-related protein 1 (RAP1), and TNF pathways.

A thorough review of ancient Traditional Chinese Medicine literature reveals that specific terms for indigestion and tonsillitis are not explicitly recorded; rather, descriptions focus on related symptoms. Chinese medicine believes that toxic heat spreads in the body first and then obstructs the throat meridian. Tonsillitis can be caused by pathogenic heat invading the lungs, the damage of kidney-YIN and the hyperactivity of deficient fire.^[31]^ It can be treated based on syndrome differentiation, with a focus on the pathogenesis. Indigestion is closely related to the heart, liver, and spleen.^[32]^ Today, unhealthy lifestyle habits such as poor diet, lack of exercise, and insufficient sleep can impair the spleen and stomach, leading to disharmony in their functions. Moreover, emotional disturbances such as anxiety and depression can cause liver Qi stagnation, which disrupts the spleen’s ability to transport and transform nutrients, resulting in insufficient nourishment and moistening of the heart. This contributes to both physical and psychological imbalances. Treatment should focus on soothing the liver, invigorating the spleen, and harmonizing the stomach for descending adverse qi and regulating qi to alleviate pain.

While TCM offers a theoretical understanding of the disease pathogenesis and treatment, modern network pharmacology provides molecular evidence supporting these principles. The “CSG-active component-target” network in the study shows that flavonoids play an important position in the network. This is consistent with the results of previous studies. For instance, Wu et al found that the alcohol extract of Iris can significantly inhibit the early and late inflammatory response of animals, and can significantly increase the respiratory tract sputum excretion of mice.^[33]^ Kim et al demonstrated that isoflavones suppressed cyclooxygenase 2 expression and prostaglandin E 2 production in cellular models.^[34]^ Other studies using imiquimod-induced psoriasis-like mice and murine colitis models further confirmed that genistein inhibits inflammation by targeting nuclear factor kappa-light-chain-enhancer of activated B cells, NOD-like receptor family, pyrin domain containing 3, and Signal Transducer and Activator of Transcription 3 pathways.^[35,36]^ Moreover, sodium tectorigenin sulfonate, a derivative of tectorigenin synthesized by Xu et al, has a 50% inhibitory concentration of 0.056 mg/mL and a therapeutic index of 53.57 on verO-E6 virus infected model cells, which is more potent than ribavirin.^[37]^ According to the anti-inflammatory, anti-allergic, antiviral, and antibacterial effects of flavonoids, it has been explained that it plays a pivotal role in the treatment of respiratory system and digestive system, but its specific pharmacological mechanism has not been fully clarified, which is the direction of future research.

In summary, from the effect of its active components, CSG has a therapeutic effect on tonsillitis and indigestion. However, the internal molecular mechanism of CSG in the treatment of these 2 diseases has not been reported. The mechanism may be related to the regulation of endogenous deoxyribonucnavase activity, nucleotide excise and repair, DNA damage recognition and other biological processes. Transcription factor proteins and enzymes are involved in the process, which is complex, but related to inflammation, immune response, and cell proliferation. Liu, Xie et al evaluated the anti-inflammatory and antioxidant effects of flavonoids, and their data suggest that flavonoids can relieve ulcerative colitis, fight gastric ulcer, improve functional dyspeptic, inhibit ileal movement and protect gastric mucosa.^[38,39]^ In addition, Xianmin et al found that flavonoids and other active ingredients form a multichannel and multi-target system in the body and exert therapeutic effects. The potential mechanism by which flavonoids alleviate chronic tonsillitis may involve reducing serum immunoglobulin E levels and inhibiting the expression of serum interleukin-4 and other inflammatory mediators.^[40]^ However, the precise mechanisms of action require further investigation.

As one of the main methods of computer aided drug design, molecular docking has been widely used in many aspects of new drug research and development. In general, the binding energy between the docking molecule and the target of AutoDock Vina is <−7.0 kJ/mol, indicating that it has strong binding activity. The top 7 chemical components (including irisin, irisin and rhamnoside) with the highest connectivity in the “active component-target of CSG” network were moleculally docked with the core genes AKT1, MAPK1, EGFR, vascular endothelial growth factor A, and PIK3R1 in the “signal gene” network. According to the docking results, the total flavonoids of CSG had good binding activity with the potential target. The analysis of the binding models between the key components and each protein showed that all the key active components entered the active sites of each protein crystal and formed hydrogen bond or hydrophobic interaction with the active sites. Therefore, it is speculated that the main components of CSG may exert therapeutic effects against tonsillitis and dyspepsia by modulating key proteins involved in the PI3K/Akt, RAP1, TNF, and other related signaling pathways. Through hydrogen bonding and hydrophobic interaction, the small molecules are well bound to the active cavities of target proteins, and the small molecules form a relatively stable complex with the protein, so it can be speculated that these small molecules are the potential active molecules of these target proteins.

In recent years, various in vivo and in vitro experimental studies have confirmed that PI3K/Akt signaling pathway plays a crucial negative regulatory role in inflammatory response, and has the ability to regulate apoptosis, proliferation, migration, differentiation, and metabolism as well as antiviral ability.^[41]^ Studies have shown that by activating the PI3K/Akt pathway, the apoptosis of gastrointestinal smooth muscle cells is inhibited and the survival of gastrointestinal smooth muscle cells is maintained.^[42]^ Chang et al found that PI3K/Akt signaling pathway mediates gastrointestinal hormone secretion and regulates gastrointestinal motility in many aspects.^[43]^ A study by Shen et al showed that intestinal afferent neurons can activate PI3K/Akt pathway to regulate the synthesis of transient receptor potential vanilloid 1 protein, which thus mediates the excitability of visceral hypersensitive neurons.^[44]^ Chen et al found that abnormal activation of PI3K can trigger and aggravate airway lesions, and inhibit the expression of EGFR and other related proteins, significantly improving the pathological status of rats.^[45]^ Li et al believed that inhibiting the expression of PI3K and related proteins can inhibit the proliferation of rat airway smooth muscle cells to a great extent.^[46]^ In endothelial cells, TNF induces a variety of intracellular signaling pathways including inflammation and immunity, which in turn increases the expression of inflammatory receptors.^[47,48]^ The TNF signaling pathway has also been confirmed to be involved in the occurrence of dyspepsia and reducing TNF can improve intestinal inflammatory in model rats. As a member of the receptor tyrosine kinase family, EGFR can mediate the clearance of apoptotic cells, which regulates innate immunity. It is closely related to autoimmune diseases. EGFR can also activate T and B lymphocytes through the mediated PI3K/Akt signaling pathway, which thus affects the immune function of macrophages and other cells.^[49]^

**Figure 10.**
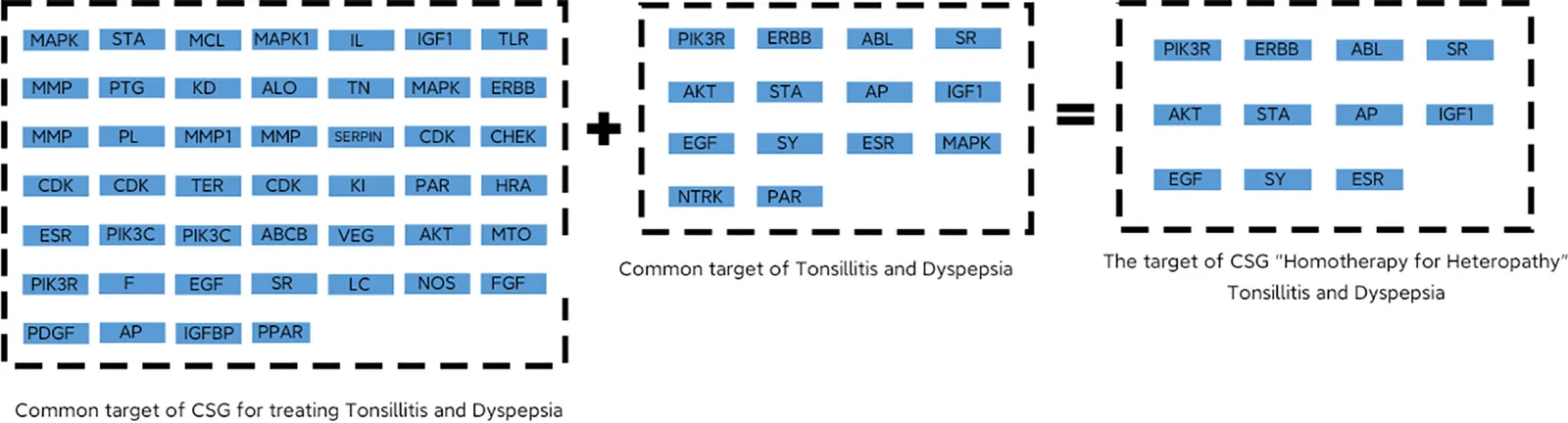
Common target of CSG for treating tonsillitis and dyspepsia.

The pathogenesis of tonsillitis and dyspepsia involves multiple biological processes in vivo. CSG may play its “co-treatment” role by regulating these biological processes through multiple targets and pathways. As shown in Figure 10, a total of 14 targets were identified for the treatment of both tonsillitis and indigestion, including AKT1, PIK3R1, and EGFR. These 14 targets share 11 common targets with the 46 core targets derived from the common target network for CSG treatment. This suggests that these 11 shared targets are likely to be the key targets involved in both conditions. Notably, the PI3K/Akt signaling pathway emerged as a central mechanism in both the shared and disease-specific networks, with AKT1 and PIK3R1 serving as major regulatory targets involved in inflammation, immunity, and cell proliferation. Molecular docking results further support that key pharmacological compounds in CSG can stably bind to AKT1, PIK3R1, and RAP1, modulating inflammation and immune responses. These findings align with the traditional Chinese medicine theory of “homotherapy for heteropathy” and provide mechanistic insights into CSG’s dual therapeutic potential. Clinically, this dual-targeting mechanism may offer valuable implications. For tonsillitis, recent guidelines emphasize the need to reduce antibiotic overuse and explore complementary therapies with anti-inflammatory and immunomodulatory effects.^[50]^ In the management of functional dyspepsia, a growing body of evidence highlights the benefits of herbal medicines (especially flavonoid-rich formulas) in relieving symptoms, enhancing gastric motility, and improving quality of life.^[51]^ Moreover, in clinical practice, CSG is especially applicable for patients with concurrent diseases or those who experience adverse reactions to medications targeting a single condition. In such cases, CSG may serve as a viable complementary or alternative therapy. Owing to its multicomponent, multi-target characteristics and its regulatory effects on shared pathological pathways, CSG holds promising potential as an integrative treatment option for both tonsillitis and functional dyspepsia.

Nevertheless, we must acknowledge several limitations and potential biases in our analysis. First, the predicted targets and pathways were obtained from public databases, which may vary in data quality depending on their release dates, update frequency, and original data sources. These factors can affect the completeness and accuracy of the results. Second, while network pharmacology and molecular docking provide valuable theoretical insights, the predicted targets and pathways have not been validated through experimental studies. In particular, molecular docking only suggests potential binding affinities and cannot confirm actual pharmacological effects. Third, the biological mechanisms of *Iris tectorum* Maxim. (CSG) remain to be elucidated through further in vitro and in vivo experiments. In future studies, we aim to verify the current findings by performing biological experiments and to explore additional key targets and pathways that may be involved in the therapeutic effects of CSG.

## 5. Conclusion

Overall, *Iris tectorum* Maxim. (CSG) has multiple functions such as inhibiting inflammation, regulating immunity and antiviral. This study is based on the traditional Chinese medicine theory of “homotherapy for heteropathy,” combined with network pharmacology and molecular docking techniques, to elucidate the mechanism of effective components of CSG for tonsillitis and dyspepsia. CSG primarily exerts its effects by modulating inflammatory factor expression and regulating immune responses through a complex network of multiple components, targets, and signaling pathways. However, these mechanisms have so far been demonstrated only at the molecular level. In the subsequent research phase, we will construct disease models by integrating in vitro cell systems with in vivo animal experiments to validate the core targets identified in this study, evaluate key clinical indicators relevant to both diseases, and explore the dose–response relationships of the active compounds. Following this, future research will focus on clinical trials to assess the therapeutic efficacy and safety of specific active components or their derivatives. Additionally, systematic pharmacokinetic and pharmacodynamic studies are necessary to elucidate the bioavailability and in vivo interactions of these compounds. This study provided a scientific basis for the further elaboration of the mechanism of “homotherapy for heteropathy” of CSG and laid the foundation of network pharmacology of the TCM theory.

## Author contributions

**Conceptualization:** Shujie Hao, Xiangming Fang.

**Data curation:** Shujie Hao, Weidong Ye.

**Funding acquisition:** Shujie Hao, Yamei Yuan, Xiangming Fang, Weidong Ye.

**Investigation:** Shujie Hao.

**Validation:** Yamei Yuan.

**Writing – original draft:** Shujie Hao.

**Writing – review & editing:** Yamei Yuan, Xiangming Fang.

## References

[1] Ye Z Lin J Wu H. Experience of Wang Mengyong in treating chronic pharyngitis from kidney [in Chinese]. Chin Inf J Tradit Chin Med. 2021;28:114–6.

[2] Masters KG Zezoff D Lasrado S. Anatomy, Head and Neck: Tonsils. StatPearls Publishing; 2025. 30969614

[3] Costa HJZR Di Francesco RC Giancoli SM de Miranda FMP Bento RF. Tonsillotomy by a fractional carbon dioxide laser: a new technique in the treatment of chronic tonsillitis. J Lasers Med Sci. 2022;13:e51. 10.34172/jlms.2022.51 PMC10082906 37041784

[4] Smith KL Hughes R Myrex P. Tonsillitis and tonsilloliths: diagnosis and management. Am Fam Physician. 2023;107:35–41. 36689967

[5] Khan DM Hamraz M Khattak AZ Ali I Khalil U Khan Z. The analysis of risk factors associated with tonsillitis in district Mardan, Pakistan. J Pak Med Assoc. 2020;70:1169–72. 10.5455/JPMA.15375 32799268

[6] Tirago TD Worku M Nima TM Ormago MD. Microbial composition, antibiotic sensitivity patterns, and contributing factors among children with tonsillitis in Hawassa Town, Sidama, Ethiopia. Int J Microbiol. 2025;2025:6366378. 10.1155/ijm/6366378 PMC12017960 40270984

[7] Haddad JD Ng P James T. Empiric treatment for acute pharyngitis. Am Fam Physician. 2019;100:713–4. 31790183

[8] Graf EH. Can rapid molecular streptococcus pyogenes testing lead to better antimicrobial stewardship for acute pharyngitis. J Appl Lab Med. 2019;4:140–2. 10.1373/jalm.2019.029983 31639658

[9] Saini N Kumar D Swarnim S Bhatt D Kishore S. Comparison of antistreptolysin O and anti-deoxyribonucleic B titers in healthy children to those with acute pharyngitis, acute rheumatic fever, and rheumatic heart disease aged 5~15years. Ann Pediatr Cardiol. 2019;12:195–200. 10.4103/apc.APC_60_18 PMC6716311 31516274

[10] Ma J Lu W. TCM master XU Jing-fan’s experience in treating chronic gastritis by method of opening-dispersing [in Chinese]. Chin J Tradit Chin Med. 2021;36:197–200.

[11] Ghoshal UC Singh R Chang FY. Epidemiology of uninvestigated and functional Indigestion in Asia: facts and fiction. J Neurogastroenterol Motil. 2011;17:235–44. 10.5056/jnm.2011.17.3.235 PMC3155059 21860815

[12] Gisbert JP. *Helicobacter pylori* and gastric disease. Med Clin (Barc). 2025;165:106974. 10.1016/j.medcli.2025.106974 40409232

[13] Cooke ZM Resciniti SM Wright BJ. Association between dietary factors, symptoms, and psychological factors in adults with dyspepsia: a cross-sectional study. Neurogastroenterol Motil. 2023;35:e14684. 10.1111/nmo.14684 37771208

[14] Amerikanou C Kleftaki SA Valsamidou E. Food, dietary patterns, or is eating behavior to blame? Analyzing the nutritional aspects of functional dyspepsia. Nutrients. 2023;15:1544. 10.3390/nu15061544 PMC10059716 36986274

[15] Chang X Zhao L Wang J Lu X Zhang S. Sini-san improves duodenal tight junction integrity in a rat model of functional indigestion. BMC Complement Altern Med. 2017;17:432. 10.1186/s12906-017-1938-2 PMC5577804 28854971

[16] Walker MM Potter MD Talley NJ. Tangible pathologies in functional dyspepsia. Best Pract Res Clin Gastroenterol. 2019;40-41:101650. 10.1016/j.bpg.2019.101650 31594648

[17] Liu B Kou Z Chen B. Effects and mechanisms of traditional Chinese medicines on functional dyspepsia: a review. Chin Herb Med. 2023;15:516–25. 10.1016/j.chmed.2023.06.001 PMC10715891 38094020

[18] Zhang J Chen T Wen Y Siah KTH Tang X. Insights and future prospects of traditional Chinese medicine in the treatment of functional dyspepsia. Phytomedicine. 2024;127:155481. 10.1016/j.phymed.2024.155481 38452693

[19] Gao B Ma Y Zhang LT Ren Q. Identification and characterization of the chemical components of *Iris tectorum* Maxim. and evaluation of their nitric oxide inhibitory activity. Rapid Commun Mass Spectrom. 2021;35:e8959. 10.1002/rcm.8959 33001505

[20] Xiong J Zhang JW. Revision of part of Chinese medicinal materials and decoction pieces in 2005 edition of pharmacopoeia of the People’s Republic of China (Part 1) [in Chinese]. Chin Inf J Tradit Chin Med. 2006;13:8–9.

[21] National Pharmacopoeia Commission. Pharmacopoeia of the People’s Republic of China, 2020 Edition 1 [in Chinese]. China Medical Science and Technology Press; 2020

[22] Chen S Yuan C Luo S. Comparison of isoflavone components in Iridis Tectori Rhizoma from different habitats [in Chinese]. J Drug Anal. 2018;38:1280–4.

[23] Li M Qian M Jiang Q Tan B Yin Y Han X. Evidence of flavonoids on disease prevention. Antioxidants (Basel). 2023;12:527. 10.3390/antiox12020527 PMC9952065 36830086

[24] Liu J Xia TR. Identification of the metabolites produced following *Iris tectorum* Maxim oral administration and a network pharmacology-based analysis of their potential pharmacological properties. Xenobiotica. 2021;51:680–8. 10.1080/00498254.2021.1907473 33779496

[25] Zhou H Zhang Y Liang H. A novel multidimensional strategy to evaluate *Belamcanda chinensis* (L) DC and *Iris tectorum* Maxim based on plant metabolomics, digital reference standard analyzer and biological activities evaluation. Chin Med. 2021;16:85. 10.1186/s13020-021-00494-3 PMC8393741 34446058

[26] Yin S Chen S Li L. Fundamental research and clinical translational application of *iris tectorum* Maxim [in Chinese]. World TCM. 2020;15:200–7.

[27] Hopkins AL. Network pharmacology: the next paradigm in drug discovery. Nat Chem Biol. 2008;4:682–90. 10.1038/nchembio.118 18936753

[28] Zhang Y Li W. Progress in network pharmacology for modern research of traditional Chinese medicine [in Chinese]. Chin J Pharmacol Toxicol. 2015;29:883–92.

[29] Zhong C Huang L Chen Y. Pharmacological mechanism of Shugan Jianpi prescription in treating diarrhea irritable bowel syndrome based on network pharmacology [in Chinese]. Chin Herb Med. 2020;43:951–60.

[30] Jaeger JM Titus BJ Blank RS. Essential anatomy and physiology of the respiratory system and the pulmonary circulation. In: Slinger P, ed. Principles and Practice of Anesthesia for Thoracic Surgery. Springer; 2019.

[31] Tian J. The clinical observation of clearing heat and benefiting pharyngeal soup in the treatment of acute and chronic pharyngitis [in Chinese]. Chin J Integr Tradit West Med. 2020;6:463–4.

[32] Wu X Gan G Li J. Effect of Shugan Jianpi Huoxue decoction on brain-gut peptides in plasma of functional dyspepsia rats with liver depression and spleen deficiency [in Chinese]. Mod Chin Appl Pharm. 2018;35:214–7.

[33] Wu Z Xiong C. A comparative study on anti-inflammatory and expectorant effects of Iris vulgaris [in Chinese]. Pharmacol Clin Tradit Chin Med. 1985;1:169.

[34] Kim YP Yamada M Lim SS. Inhibition by tectorigenin and tectoridin of prostaglandin E2 production and cyclooxygenase-2 induction in rat peritoneal macrophages. Biochim Biophys Acta. 1999;1438:399–407. 10.1016/s1388-1981(99)00067-0 10366782

[35] Wang A Wei J Lu C. Genistein suppresses psoriasis-related inflammation through a STAT3-NF-κB-dependent mechanism in keratinocytes. Int Immunopharmacol. 2019;69:270–8. 10.1016/j.intimp.2019.01.054 30743203

[36] Chen Y Le TH Du Q. Genistein protects against DSS-induced colitis by inhibiting NLRP3 inflammasome via TGR5-cAMP signaling. Int Immunopharmacol. 2019;71:144–54. 10.1016/j.intimp.2019.01.021 30901677

[37] Xu X. Structural modification of iris aglycone and its anti-SARS activity in vitro [in Chinese]. Proceedings of the 3rd International Conference on Modernization of Chinese Medicine, Chengdu: 2010.

[38] Liu D Yu X Sun H Zhang W Liu G Zhu L. Flos lonicerae flavonoids attenuate experimental ulcerative colitis in rats via suppression of NF-κB signaling pathway. Naunyn Schmiedebergs Arch Pharmacol. 2020;393:2481–94. 10.1007/s00210-020-01814-4 32125461

[39] Xie W Liu G. Effect of flavone on gastrointestinal motility in functional dyspepsia rats [in Chinese]. China Prescr Drugs. 2016;14:23–4.

[40] You X Zou G Zhao J. Effects of the extract of Rhizoma spp. on the expression of interleukin-4 and immunoglobulin E in serum of rabbits with chronic pharyngitis [in Chinese]. China Pharm. 2016;25:3–4.

[41] Wang ZG Wang Y Huang Y. bFGF regulates autophagy and ubiquitinated protein accumulation induced by myocardial ischemia/reperfusion via the activation of the PI3K/Akt/mTOR pathway. Sci Rep. 2015;5:9287–90. 10.1038/srep09287 PMC4365411 25787015

[42] An X Long C Deng X. Higenamine inhibits apoptosis and maintains survival of gastric smooth muscle cells in diabetic gastroparesis rat model via activating the beta2-AR/PI3K/AKT pathway. Biomed Pharmacother. 2017;95:1710–7. 10.1016/j.biopha.2017.08.112 28958133

[43] Chang X Zhao L Wang J Lu X Zhang S. Sini-san improves duodenal tight junction integrity in a rat model of functional dyspepsia. BMC Complement Altern Med. 2017;17:432. 10.1186/s12906-017-1938-2 PMC5577804 28854971

[44] Shen S Al-Thumairy HW Hashmi F Qiao L-Y. Regulation of transient receptor potential cation channel subfamily V1 protein synthesis by the phosphoinositide 3-kinase/Akt pathway in colonic hypersensitivity. Exp Neurol. 2017;295:104–15. 10.1016/j.expneurol.2017.06.007 PMC5535076 28587873

[45] Chen Y Feng G Li G. Effect of Jiduqingfei Heji on EGFR/MAPK signal transduction pathway in chronic obstructive pulmonary disease model rats with airway mucus hypersecretion [in Chinese]. Chin J Tradit Chin Med. 2016;31:1996–2001.

[46] Li M Chen Y Cai R. Effects of Wenshen Yiqi granules on airway smooth muscle cell proliferation and PI3K/AKT/mTOR signaling pathway expression in rats with chronic obstructive pulmonary disease [in Chinese]. Chin J Tradit Chin Med. 2019;34:1711–4.

[47] Pan HH Hsiao YP Chen PJ. Epithelial growth factor receptor tyrosine kinase inhibitors alleviate house dust mite allergen Der p 2-induced IL-6 and IL-8. Environ Toxicol. 2019;34:476–85. 10.1002/tox.22701 30623574

[48] Singh B Kosuru R Lakshmikanthan S. Endothelial rap1 (ras-Association proximate 1) restricts inflammatory signaling to protect from the progression of atherosclerosis. Arterioscler Thromb Vasc Biol. 2021;41:638–50. 10.1161/ATVBAHA.120.315401 PMC8105264 33267664

[49] An Z Aksoy O Zheng T Fan Q-W Weiss WA. Epidermal growth factor receptor and EGFRvIII in glioblastoma: signaling pathways and targeted therapies. Oncogene. 2018;37:1561–75. 10.1038/s41388-017-0045-7 PMC5860944 29321659

[50] Kuijpers SME Buis DTP Ziesemer KA. The evidence base for the optimal antibiotic treatment duration of upper and lower respiratory tract infections: an umbrella review. Lancet Infect Dis. 2025;25:94–113. 10.1016/S1473-3099(24)00456-0 39243792

[51] Heiran A Bagheri Lankarani K Bradley R Simab A Pasalar M. Efficacy of herbal treatments for functional dyspepsia: a systematic review and meta-analysis of randomized clinical trials. Phytother Res. 2022;36:686–704. 10.1002/ptr.7333 34851546

